# Projected declines in global DHA availability for human consumption as a result of global warming

**DOI:** 10.1007/s13280-019-01234-6

**Published:** 2019-09-12

**Authors:** Stefanie M. Colombo, Timothy F. M. Rodgers, Miriam L. Diamond, Richard P. Bazinet, Michael T. Arts

**Affiliations:** 1 Present Address: Department of Animal Science and Aquaculture, Faculty of Agriculture, Dalhousie University, 58 Sipu Road, Haley Building, Bible Hill, Truro, NS B2N 5E3 Canada; 2 Department of Chemical Engineering and Applied Chemistry, University of Toronto, Toronto, ON Canada; 3 Department of Earth Sciences, University of Toronto, 22 Russell St., Toronto, ON M5S 3B1 Canada; 4 Department of Nutritional Sciences, University of Toronto, Medical Sciences Building, 5th Floor, Room 5358, 1 King’s College Circle, Toronto, ON M5S 1A8 Canada; 5 Department of Chemistry and Biology, Ryerson University, 350 Victoria St., Toronto, ON M5B 2K3 Canada

**Keywords:** Aquaculture, Climate change, Docosahexaenoic acid (DHA), Fisheries, Global warming

## Abstract

Docosahexaenoic acid (DHA) is an essential, omega-3, long-chain polyunsaturated fatty acid that is a key component of cell membranes and plays a vital role in vertebrate brain function. The capacity to synthesize DHA is limited in mammals, despite its critical role in neurological development and health. For humans, DHA is most commonly obtained by eating fish. Global warming is predicted to reduce the de novo synthesis of DHA by algae, at the base of aquatic food chains, and which is expected to reduce DHA transferred to fish. We estimated the global quantity of DHA (total and per capita) currently available from commercial (wild caught and aquaculture) and recreational fisheries. The potential decrease in the amount of DHA available from fish for human consumption was modeled using the predicted effect of established global warming scenarios on algal DHA production and ensuing transfer to fish. We conclude that an increase in water temperature could result, depending on the climate scenario and location, in a ~ 10 to 58% loss of globally available DHA by 2100, potentially limiting the availability of this critical nutrient to humans. Inland waters show the greatest potential for climate-warming-induced decreases in DHA available for human consumption. The projected decrease in DHA availability as a result of global warming would disproportionately affect vulnerable populations (e.g., fetuses, infants), especially in inland Africa (due to low reported per capita DHA availability). We estimated, in the worst-case scenario, that DHA availability could decline to levels where 96% of the global population may not have access to sufficient DHA.

**Electronic supplementary material:**

The online version of this article (10.1007/s13280-019-01234-6) contains supplementary material, which is available to authorized users.

## Introduction

Humans are the causative agents in a climate change experiment of global proportions with serious, life-altering consequences that are not yet wholly understood. Reacting to the copious volumes of scientific evidence, climate change has recently been called, by world leaders and other notable public figures (e.g., US President Barak Obama in his State of the Union Address 2015; BBC News 2018), “*The Greatest Threat to Future Generations.”* In its 2018 report, the Intergovernmental Panel on Climate Change (IPCC 2018) assigned a high level of confidence to predictions that failing to reduce our greenhouse emissions to levels commensurate with a global surface air temperature increase of 1.5 °C or less would, among other things (e.g., sea level rise, loss of ice, drought in some areas and excess rain in other areas), increase the probability of exacerbating, (a) negative impacts on biodiversity and ecosystems, including species loss and extinction, (b) increase in ocean acidity and decrease in ocean oxygen levels, and (c) increased risks to marine biodiversity, fisheries, and ecosystems, and their functions and services to humans.

We are only beginning to understand the effects of global climate-change-induced increases in temperature on critical underlying biochemical reactions. Chemistry drives biological systems and core chemical reactions (e.g., photosynthesis, Kreb’s Cycle) that are known to be sensitive to changes in ambient temperature, particularly in ectotherms. Another core biochemical suite of reactions are the synthesis reactions that produce the compounds (fatty acids) that make up the bulk of plant and animal cell membranes, i.e., the chemical partitions that separate life from non-life. The composition of fatty acids in the cell membranes of plants and ectothermic animals is known to be affected by ambient temperature. This is because many ectotherms (including algae that fuel life in the world’s waters) adapt to changing temperatures by altering the proportions of saturated vs unsaturated fatty acids in their cell membranes in order to maintain an optimal level of fluidity (Guschina and Harwood 2006; Arts and Kohler 2009); a process known as homeoviscous adaptation (Sinensky 1974). Specifically, the multiple double bonds in polyunsaturated fatty acids (PUFA) contribute to increased membrane fluidity and hence, as temperatures fall, algal cells produce more PUFA to counteract the tendency of cell membranes to become rigid. In contrast, acclimating to increasing temperature involves decreasing PUFA membrane content and increasing the proportion of saturated fatty acids, both of which contribute to making cell membranes more rigid.

One of the PUFA involved in the response of homeoviscous adaptation is docosahexaenoic acid (DHA; 22:6n-3); an omega-3 (n-3) long-chain polyunsaturated fatty acid (LC-PUFA). Thus, an important, and as yet not widely appreciated, threat posed by global warming is that increasing water temperatures, through the mechanism of homeoviscous adaptation, is expected to reduce the global production of DHA in aquatic ecosystems. For example, Hixson and Arts (2016) using regression models predicted that, in response to a 2.5 °C increase in surface water temperature predicted by the year 2100 (IPCC 2014), algae could potentially reduce DHA synthesis by up to 28% globally. Since algae are the principle suppliers of DHA to higher trophic levels (e.g., fish), a reduction in the synthesis of DHA by algae as water temperatures rise has the potential to translate into a profound impact on the total supply of DHA to aquatic ecosystems, and hence the availability of DHA to humans.

DHA is essential for vertebrate brain development and function. In fact, the availability of DHA through fish consumption has been hypothesized to have allowed for hominid brain development and increased cognitive ability during hominid evolution (Crawford 1992; Joordens et al. 2014). It is the most abundant PUFA in the mammalian brain (> 10% of brain fatty acids; Makrides et al. 1994), where it regulates many physiological processes including neuroprotection, cell survival, and inflammation (Alessandri et al. 2004; Bazinet and Layé 2014). It is also intimately involved in neural cell growth and differentiation, and in neuronal signaling. DHA is also highly accumulated in the central nervous system and retina, and as such, is thought to be essential for optimal development of these regions (Rogers et al. 2013).

Observational and meta-analyses have found associations between indicators of maternal DHA status and infant and child cognitive, physiological, and behavioral outcomes (e.g., Carlson 2009) indicating that a supply of DHA is particularly important for the fetus, and infant (Forsythe et al. 2016; Stark et al. 2016). The absolute and relative amounts of DHA in the brain increase during fetal development, especially in the last trimester of pregnancy (Cunnane et al. 2000; Kuipers et al. 2012). DHA is especially important for optimal development during the first 6 months of life postpartum (Lauritzen et al. 2016). In addition, DHA supplementation during late pregnancy and early infancy has been found to subtly improve infant neural development (Shulkin et al. 2018). Although its precise role in brain functioning and cognition throughout life has not yet been fully defined or quantified, the high accretion rates (Kris-Etherton et al. 2009) and the amount of DHA accumulated in the developing brain suggest that DHA is a vital component in central nervous system function. This has led numerous agencies to recommend DHA intake in infants (e.g., 100 mg/day by the Food and Agriculture Organization of the United Nations; FAO 2010).

Despite its importance, the capacity to synthesize DHA de novo is limited in the developing fetal and infant brain (Cunnane et al. 2000) leading to the dependence on the mother, and, in infants, on a combination of the mother and the diet, in order to obtain sufficient DHA for optimal brain development. As de novo synthesis is also limited in adults, a dietary source is again the most efficient method of acquiring DHA. Sources include DHA-enriched eggs from hens consuming alpha-linolenic acid (ALA)-rich flaxseed (Lewis et al. 2000) (which they can convert to DHA with reasonable efficiency), and in grass-grazing ruminant animals that can convert a small proportion of ALA to DHA, which is stored in the meat and milk (Wood et al. 1999; Petit et al. 2002). Poultry meat provides the largest potential DHA intake of these sources (Givens 2009). However, livestock that are fed diets that are high in cereal grains do not produce as much DHA compared to pasture feeding ruminants. The problem is that, in recent years, ruminants are more often fed grain-based diets (and which contain lower amounts of n-3 FA), due to increased forage prices and land use competition (Provenza et al. 2019), which then lowers the DHA supply in meat for consumers. There have also been efforts to produce DHA by other means, such as microbial and microalgae production (Sprague et al. 2017; Tibbetts 2018) and transgenic crops (Napier et al. 2019), although there is uncertainty around the future development and availability of these sources. However, overall, DHA is currently most effectively obtained by eating fish and seafood, and/or taking fish and/or algal oil supplements (Arts et al. 2001; Calder 2015). DHA is produced in aquatic environments primarily by algae at the base of the food web and is successively consumed and retained by higher trophic level organisms, notably fish (Kainz et al. 2004; Colombo et al. 2017). As terrestrial plants do not naturally produce DHA (Colombo et al. 2017), fish and other types of seafood (both wild and cultured) are the main sources of DHA to the human population; and this is the focus of our study.

To compound concerns over DHA availability due to the reduced production expected because of global warming, overfishing has stagnated capture fisheries production over the past five years as a consequence of the increased human demand due to population growth (FAO 2018). This has led Golden et al. (2016) to estimate that, driven by declines in capture fisheries, 10% of the global human population could face low DHA and other micronutrient intakes over the coming decades. Our goal was to explore the potential impact of reduced DHA availability for human consumption due to global warming. In this analysis, we draw attention to yet another unintended consequence of global warming; one which we predict will have a disproportionate impact on already-vulnerable populations. We consider both wild caught and fish from aquaculture in our analysis and sources of uncertainty in our projections.

## Materials and Methods

### Model development

Projections of the global distribution and future trends of fisheries and aquaculture production are highly uncertain (e.g., Blanchard et al. 2012; Cheung et al. 2016). To cope with this uncertainty, a bottom-up approach to estimate fisheries nutrient production based on detailed models of spatially discrete ocean productivity and fish distributions can be adopted (e.g., Fernandes et al. 2013; Jennings and Collingridge 2015). However, this approach still leaves considerable uncertainty and may be limited to certain pre-selected species and regions, and/or may exclude freshwater fisheries and aquaculture (Nielsen et al. 2018). Alternatively, a top-down approach based on large-scale reported production or consumption data could be used (e.g., Smith et al. 2016). We followed this approach and developed a top-down model to estimate the global amount of DHA from marine and freshwater (inland) capture fisheries and aquaculture available for human consumption now and in 2100, based on current (*T*_1_, the “base case”) and future water temperatures (*T*_2_). Our model consists of two components: to predict the, (a) annual global production of DHA, and (b) change in DHA production as a function of temperature. Throughout our modeling, we quantified the uncertainty associated with each input parameter, allowing us to estimate model output uncertainty.

The model to predict the global annual production of DHA (*ΣM*_DHA_, tonnes year^−1^) from fish available for human consumption in each Food and Agriculture Organization (FAO) of the United Nations fishing zone *i* in the base case was

1 $$ \varSigma M_{{{\text{DHA}},i}} = \, M_{{{\text{Fish}},i}} \cdot \, F_{\text{Fillet}} \cdot \, F_{{{\text{Lipid}},i}} \cdot \, F_{{{\text{DHA}},i}}, $$

where *M*_Fish,*i*_ is the sum of fish production (aquaculture and capture) in zone *i* (tonnes year^−1^); *F*_Fillet_ is the fraction of fillet yield (edible portion) from whole fish, summed for all fish species; *F*_Lipid,*i*_ is the fraction of total lipid in fillet; and, *F*_DHA,*i*_ is the fraction of DHA in total lipid in the fillet, calculated as the area-weighted average within each latitudinal band of each FAO fishing zone *i.*

The change in mass of DHA in fish produced annually between temperatures *T*_1_ and *T*_2_, ∆*M*_DHA,*i*_ (tonnes year^−1^), was estimated for each FAO zone *i* as

2 $$ \Delta M_{{{\text{DHA}},i}} = M_{{{\text{DHA}},i}} \cdot m \cdot \left( {T_{ 2,i\,} - \,{\text{T}}_{ 1,i} } \right), $$

where *m* is the change in algal DHA content (%) per °C, which we obtained by modifying the linear regression of Hixson and Arts (2016) relating water temperature to algal DHA content. Full details of all terms in Eqs. 1 and 2 are available in the Supplementary Data File S2. We show *ΣM*_DHA,*i*_ and ∆*M*_DHA,*i*_ results for each of the four representative climate pathway (RCP) scenarios (i.e., RCP 2.6, 4.5, 6.0, 8.5) generated by the IPCC. These four scenarios give a range of water temperature increases and corresponding *ΣM*_DHA,*i*_ and ∆*M*_DHA,*i*_ results depending on human responses to limit climate change.

### Model parameterization

The amount of DHA from capture and aquaculture fish production (*M*_Fish,*i*_) was estimated for each fishing zone (both marine and freshwater) assigned by the FAO (2016; see Supplementary Data “Model Output Summary” Table S1). We used global marine fisheries catch reconstruction data from the *Sea Around Us* project (Pauly and Zeller 2015) along with global inland fisheries catch and aquaculture production data from the FAO, as the *Sea Around Us* database does not include catch reconstructions for the inland fishing zones or estimates of aquaculture production. We multiplied the FAO inland catch data by a factor of 1.0–3.7 (see SI for full details) to account for the known under-reporting of subsistence and artisanal fisheries in FAO-reported landings data (Pauly and Zeller 2015; FAO 2016; Pauly and Zeller 2016) .

We used the fillet fraction of all fish species in all FAO zones (FAO 1989) to estimate *F*_Fillet_. To parameterize *F*_Lipid,*i*_ and *F*_DHA,*i*_, we used data reported by Hixson et al. (2015) and Colombo et al. (2017). Most FAO zones spanned large latitudinal ranges over which *F*_Lipid_ and *F*_DHA_ in fish vary. Thus, we defined latitudinal bands by the locations of the tropics and the polar regions (i.e., as ~ 23° N/S for the tropics and ~ 66°N/S for the polar zones; Laskar 1986), and defined *F*_Lipid_ and *F*_DHA_ for each of these bands. We defined *F*_Lipid,*i*_ and *F*_DHA,*i*_ using the area-weighted average of these latitudinal bands within each FAO fishing zone *i*.

We projected temperature increases from 2010 (*T*_1_) to 2100 (*T*_2_) in order to match the IPCC AR5 scenarios (Hoegh-Guldberg et al. 2014). Projected seawater temperature increases by year 2100 were based on the representative concentration pathway (RCP) predictions estimated by IPCC AR5, namely RCP 2.6, RCP 4.5, RCP 6.0, and RCP 8.5 (Hoegh-Guldberg et al. 2014; IPCC 2014). Projected increases in freshwater temperatures over the same time-period were estimated using the National Center for Atmospheric Research (NCAR) Community Climate System Model (CCSM) surface skin temperature projections in each of the FAO inland zones (NCAR 2004). We obtained these data at a variety of spatial scales and then averaged them across each FAO fishing zone to give the average zonal increase in temperature between 2010 and 2100.

Finally, we used the United Nations (UN) World Population Prospects 2017 medium variant (UN 2017) to calculate an annual per capita estimate of available DHA in the base case and in 2100 (g DHA per capita) under each IPCC scenario. We assigned the putative DHA available in each FAO fishing zone to the country of the fishing fleet (Pauly and Zeller 2015; FAO 2016), as precise estimates of international trade flows are beyond the scope of this paper (see Supplementary Data Table S1 “DHA Allocation” for full details).

### Model assumptions

The model relies on several assumptions. First, we assumed that the temperature-dependent change in DHA content in algae predicted by a modified linear regression model of data reported by Hixson and Arts (2016) would be propagated through to capture and aquaculture fish, and that this simple relationship would be equivalent in all fish in all FAO zones. The regression model was based on a database of > 1,000 observations of algae broadly representative of DHA-producing algal species across the world, considering the production of DHA by algae associated with the impact of increasing water temperature.

This assumption implies bottom-up control of DHA levels in marine and freshwater systems. DHA has been shown to be efficiently transferred from algae, up the food chain, to fish where it is generally highly retained (Hixson et al. 2015; Colombo et al. 2017). In zooplankton, heat stress lowered the DHA concentration in copepods regardless of the resources available, and this implies negative effects for higher trophic levels (Werbrouck et al. 2016). Further, studies have shown that variability in the PUFA content of freshwater phytoplankton communities produces variability in the PUFA content of fish (Ahlgren et al. 1996), and the fatty acid composition of marine fish is well known to reflect the fatty acid content of their diet, and, ultimately, of local phytoplankton (St. John and Lund 1996).

The ability of fish to synthesize DHA (from omega-3 precursors, including ALA and EPA), into DHA, is thought to be low and therefore we did not account for this in our model. Generally, when fish consume a diet without DHA, their own synthesis capabilities are not sufficient to meet their own requirements (Tocher 2015). Freshwater fish are thought to have a better ability to synthesize DHA than marine fish, due to the difference in DHA production between freshwater and marine ecosystems (Leaver et al. 2008). However, this level of DHA synthesis has not been accurately quantified in most fish species, other than salmonids (e.g., Bell et al. 2001; Sanden et al. 2011). For salmonids, it is possible that DHA synthesis in fish may compensate in some way for reduced diet DHA availability, acting as a net producer of DHA (Sanden et al. 2011; Sawyer et al. 2016). However, algae are also expected to produce fewer DHA precursors in response to warming waters particularly ALA (Fuschino et al. 2011; Hixson and Arts 2016), which means less available substrate for DHA synthesis in fish (Tocher 2003). Further to this point, although salmonids have relatively high amounts of DHA in the fillet (e.g., 0.8–1.3 g per 100 g fillet), their nutritional requirement for survival is 1–2% (DHA plus EPA) of total lipid intake (Tocher 2010). Therefore, it is very unlikely that the de novo synthesis of salmonids would extend higher than needed for survival, if under dietary deficiency.

Second, we assumed that the same assemblages of species that were found in each FAO zone in the base case (in 2010) were still there in year 2100. Changes in species assembly are difficult to predict quantitatively and require modeling that was beyond the scope of this paper (e.g., Cheung et al. 2016). However, we address this through our uncertainty analysis, which provides estimates of DHA production (*M*_DHA_) in 2100 that account for the variation of fish species in each of the FAO zones by changing *F*_Lipid,*i*_ and *F*_DHA,*i*_.

Third, we assumed that fish production (capture plus aquaculture) was constant between the 2010 base case and year 2100. Projections of future fish catch are known to be highly uncertain (e.g., Cheung et al. 2016; Golden et al. 2016), and total fish production may be maintained even as wild fish catch falls depending on aquaculture production (Froehlich et al. 2018). As such, we considered this in our uncertainty analysis by varying the magnitudes of total fish catch and aquaculture production separately.

Fourth, the main dietary source of DHA for farmed fish is from fish oil, harvested from capture fisheries. As we estimate in our model, wild fish will have a reduced level of DHA which will subsequently reduce the DHA available to farmed fish. However, the aquaculture industry has been working, for environmental and economic reasons, to reduce the level of fish oil in commercial fish feeds. These two factors may potentially reduce the availability of dietary DHA for farmed fish, and subsequent storage of DHA in the fillet (Sprague et al. 2016). Although not yet market ready, other sources of DHA, such as transgenic camelina oil, microalgae, or microbial oils may soon become available to replace fish oil in feeds for farmed fish (Osmond and Colombo 2019). We did not account for the contribution of wild fish to farmed fish in the model, although currently aquaculture is a major user of fish meal and fish oil. This omission may have a compounding effect on estimates of global DHA production which is very complex and uncertain. It is also possible that aquaculture will use other sources of DHA in the near future.

### Model uncertainty and sensitivity

Noting the numerous uncertainties and assumptions in the model, the estimates of *M*_DHA, base case_, *M*_DHA,2100_, and ∆*M*_DHA_ were subjected to Monte Carlo Simulation (MCS) uncertainty analysis to characterize the uncertainty bounds in the calculation by estimating the empirical probability density function of the model outputs. We performed one million trials in Microsoft Excel using Oracle Crystal Ball (Oracle Corporation, Redwood Shores, CA, USA; version 11.1). This analysis also identified model sensitivity to the input parameters or groups of input parameters contributing the most to the uncertainty of the model.

Where sufficient data were available, we parameterized the probability density function for each input variable using the Oracle Crystal Ball Distribution fitting tool, which selects a probability distribution shape and parameters (e.g., standard deviation and location for a log-normal distribution) by minimizing the value of the Anderson–Darling test statistic (Anderson and Darling 1952). Where this was not possible, we assigned a probability density function based on professional judgment. The SI contains descriptions of each parameter used in the model, along with the probability distributions for each input variable and model output. The uncertainty in the temperature projections was quantified using the IPCC RCPs, which give a range of possible temperatures in 2100, depending on the efficacy of human mitigation efforts.

Global model sensitivity was calculated using the Spearman’s rank correlation coefficient, a non-parametric test of correlation between datasets, which correlates the rank-transformed values of the model outputs with the rank transformed values of the model inputs. A value of + 1 or − 1 indicates a perfectly monotonic relationship between the model inputs and the model outputs. We present the normalized rank regression coefficient or NRRC (Iman and Conover 1979), where the Spearman’s rank sensitivity is normalized to the sum of the absolute sensitivity values for each model output. This provides an estimate of the relative contribution to the total uncertainty of the output from each of the inputs (Manache and Melching 2008) and is appropriate for non-linear, monotonic models (Marino et al. 2008).

## Results

### Model results

Results are presented as the median value and 90% confidence interval obtained from the Monte Carlo analysis (e.g., Tables 1 and 2). The probability distributions of *M*_DHA,*i*_, *ΣM*_DHA, base case_, *ΣM*_DHA,2100_, and ∆*M*_DHA_ are presented in Supplementary Data File (“Report” Table S1).

**Table 1:** Current (base case) and year 2100 estimates of *ΣM*_DHA_ according to the IPCC RCP scenarios. Values listed are median *∑M*_DHA_ from marine and freshwater capture fisheries and aquaculture (median × 10^3^ tonnes in bold, with 90% confidence interval). Note that subtotals do not equal the overall total, as each output was analyzed independently

	Base case			RCP 2.6			RCP 4.5			RCP 6.0			RCP 8.5		
	DHA	5%	95%	DHA	5%	95%	DHA	5%	95%	DHA	5%	95%	DHA	5%	95%
Freshwater	**100**	18	590	**94**	16	550	**72**	12	420	**59**	9.6	360	**20**	0.0	180
Marine	**250**	73	960	**220**	65	850	**190**	54	720	**170**	48	650	**110**	25	450
Total	**370**	110	1,500	**330**	96	1,400	**270**	78	1,100	**240**	66	970	**140**	27	610

**Table 2:** The change in DHA, ∆*M*_DHA_ (tonnes year^−1^, in bold) and percentage change at 5 and 95% confidence limits, from the base case to year 2100 according to the IPCC representative climate pathways

	RCP 2.6			RCP 4.5			RCP 6.0			RCP 8.5		
	DHA	5%	95%	DHA	5%	95%	DHA	5%	95%	DHA	5%	95%
Freshwater	**7.0**	1.2	42	**28**	4.7	170	**39**	6.7	240	**73**	12	440
Marine	**25**	6.4	120	**58**	15	260	**76**	20	340	**130**	34	560
Total	**34**	8.7	160	**90**	23	410	**122**	31	560	**210**	55	970
Proportional decrease	**9.2%**	8.2%	11%	**25%**	22%	29%	**33%**	30%	40%	**58%**	52%	68%

#### Current global DHA contribution from fish, *M*_DHA, base case_

The estimated current total global amount of DHA from fish available for annual human consumption, *ΣM*_DHA,base case_, was calculated as a median of 370 × 10^3^ tonnes (110 × 10^3^–1500 × 10^3^ 90% confidence interval). Freshwater fisheries supplied 100 × 10^3^ (18 × 10^3^–590 × 10^3^) tonnes and marine fish supplied 250 × 10^3^ (73 × 10^3^–1000 × 10^3^) tonnes, respectively, of the global catch (Table 1).

**Fig. 1:**
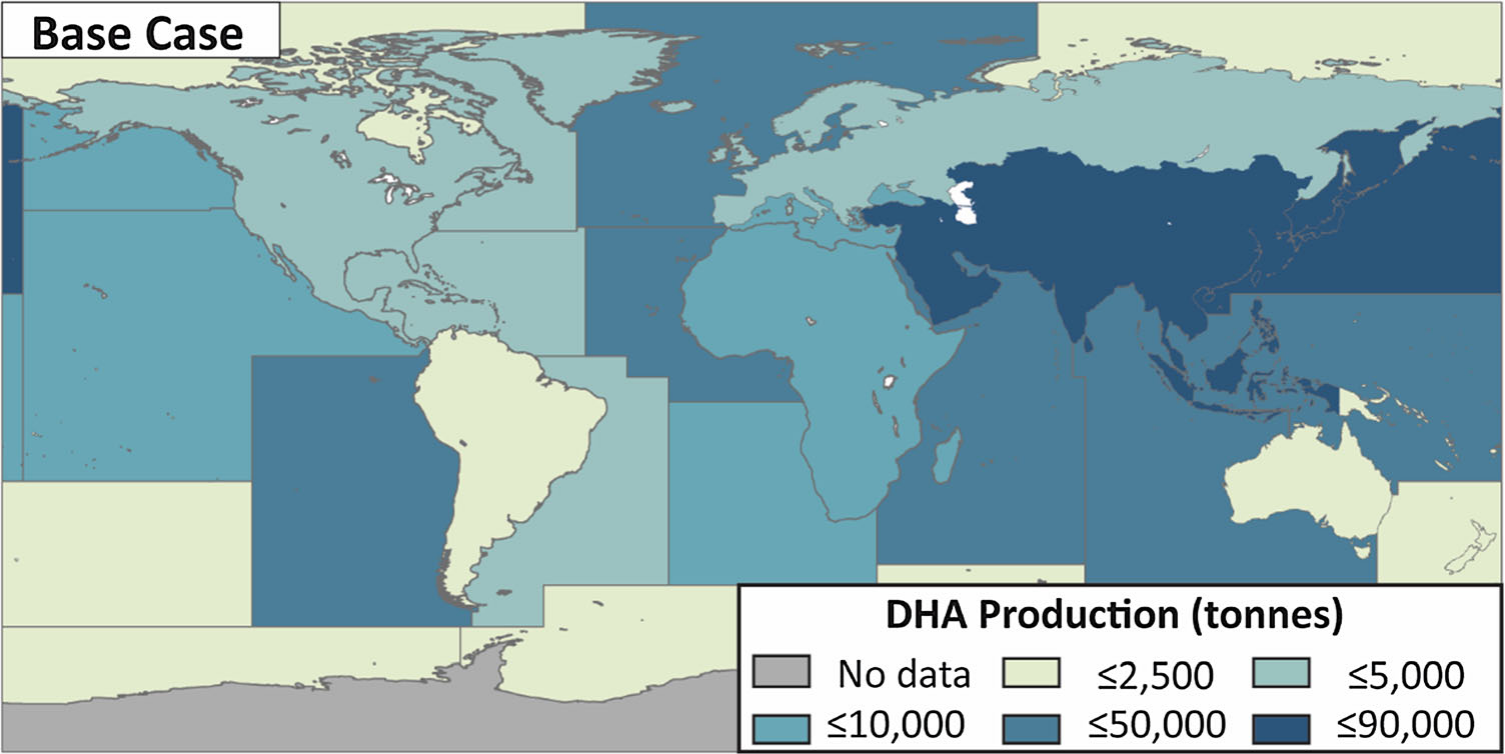
The amount of DHA available from fish for human consumption (*M*_DHA_, tonnes year^−1^) in the base case, represented by fishing zone

The largest contribution to *ΣM*_DHA, base case_ was from the freshwater fisheries of Asia (Fig. 1), driven by large fish production from both aquaculture and capture fisheries. Marine fisheries of the Pacific Northwest were the next largest source, in this case driven by the largest reconstructed fish catch among all of the FAO zones. The South American inland zone, with low reported catches and mostly tropical (and therefore lower) estimated F_Lipid,i_ and F_DHA,i_, had low production, along with the low-catch polar regions. Full results of *M*_DHA,i_ for the base case by zone are available in the Supplementary Data File S2.

#### Predicted global DHA from fish under climate change scenarios

Under the RCP 8.5 (business as usual) scenario, we estimated *ΣM*_DHA,2100_ at 140 × 10^3^ (27 × 10^3^–610 × 10^3^) tonnes (Table 1). This represents an average decrease in DHA availability from fish for human consumption of 58% (51–68%) globally (Table 2), with even larger decreases in some regions as explored below. Full data details of *ΣM*_DHA,2100_ and ∆*M*_DHA_ by FAO catch zone for each RCP are available in the Supplementary Data File (“Model Output Summary Data” Table S1).

**Fig. 2:**
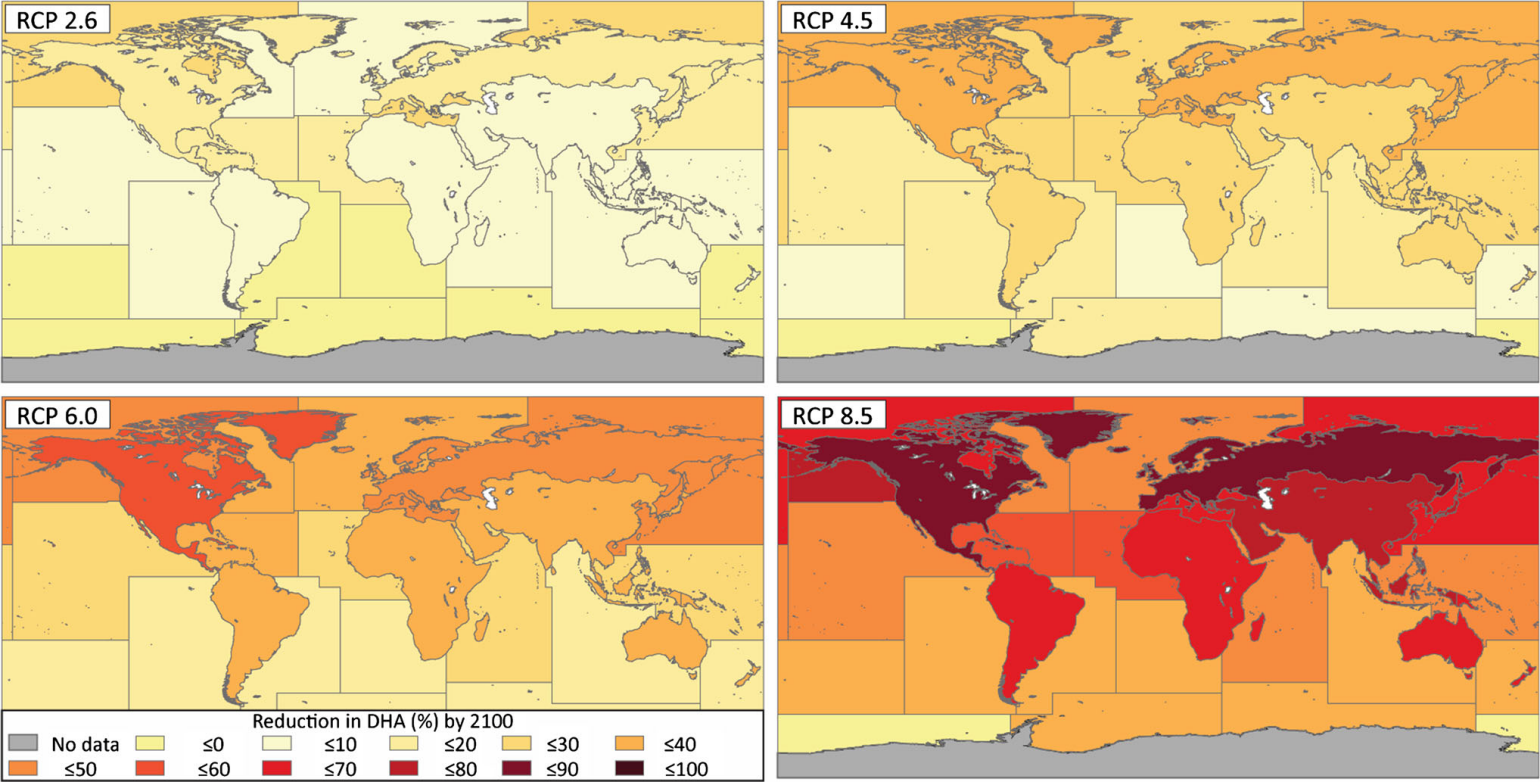
Change in the amount of DHA available from fish for human consumption (∆*M*_DHA_, tonnes year^−1^) under each of the IPCC representative concentration pathways (RCP): **a** RCP 2.6, **b** RCP 4.5, **c** RCP, 6.0, and **d** RCP 8.5

Geographic differences in *ΣM*_DHA,2100_ declines, expressed as a percentage, decrease from the 2010 base case year to 2100 and according to each FAO zone under each climate change scenario, are illustrated in Fig. 2 (data from “Model Output Summary Data” Table S1 in Supplementary Data File).

The greatest relative decrease in DHA availability for marine fisheries under RCP 8.5 occurred in the Arctic sea, where we predicted a 70% (67–82%) decline, along with a 71% (67–79%) decline in the Pacific Northeast. Our predictions showed that the high-latitude freshwater zones of Europe of 86% (83–90%) and North America of 86% (82–90%) will have the highest percentage decreases overall, due to greater projected warming in Arctic and inland waters that experience greater temperature extremes than marine waters. The largest absolute ∆*M*_DHA_ occurred in the Asian inland fishery, with a decline of 61 × 10^3^ (8.9–400 × 10^3^) tonnes under RCP 8.5, equivalent to almost a fifth of *ΣM*_DHA,base case_. Overall, the freshwater fishing zones showed higher relative declines in DHA than the marine zones under every scenario except for RCP 2.6.

#### Predicted DHA per capita

**Fig. 3:**
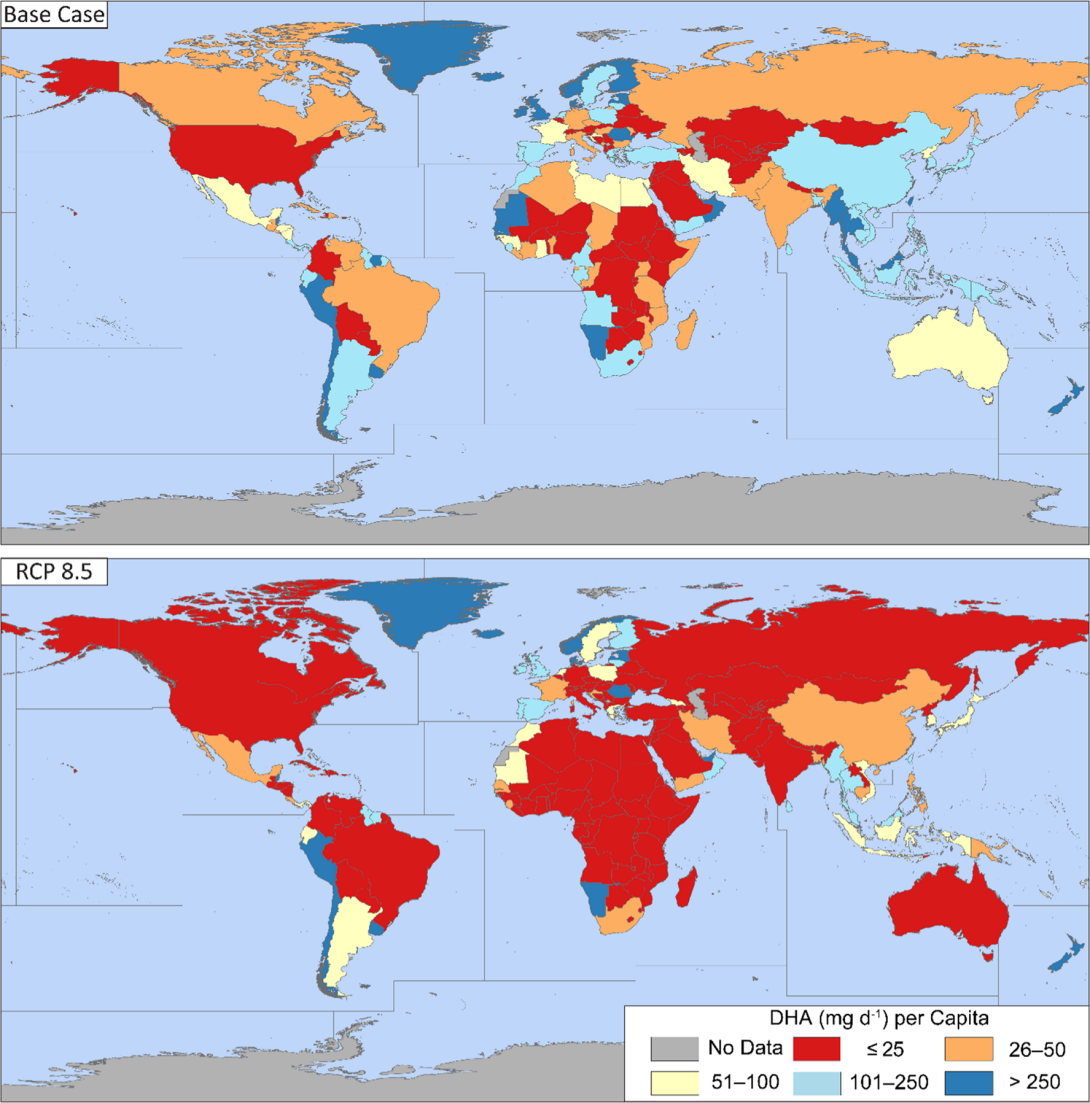
DHA production (mg day^−1^) per capita in a single fishing year for the base case and by 2100, under RCP 8.5. Fig. S1 shows the DHA per capita under the other IPCC scenarios by 2100. Population estimates are from the median variant of the UN World Population Prospects (2017). Political boundaries base map from https://gadm.org/

Next, we investigated the possible implications for reduced global DHA production on individual (per capita) consumption from DHA sourced from wild and farmed fish (Fig. 3). Recommendations for DHA dietary intake can vary greatly. The FAO recommends ~ 100 mg day^−1^ DHA for infants aged 0–24 months (FAO 2010). Other organizations worldwide, such as the World Health Organization and the American Dietetic Association, recommend ~ 500 mg day^−1^ DHA plus EPA for adults, or ~ 250 mg day^−1^ of DHA alone, while others have suggested that 50 mg day^−1^ DHA would suffice (Cunnane et al. 2000; Kris-Etherton et al. 2009). The most recent consensus suggests that 250 mg day^−1^ of DHA (plus EPA) is recommended for adults (Rimm et al. 2018; Zhang et al. 2018).

Countries with large fish production and relatively low populations, such as Greenland, Norway, Chile, and New Zealand remained above 250 mg day^−1^ per capita in both the base-case scenario and under RCP 8.5. The population of Norway is projected to increase 1.6-fold by 2100 while we predict that their DHA harvest will decline by 54%, so the continued domestic surplus of DHA is due to the large Norwegian fish industry, which currently takes 35% of the harvest in the North Atlantic FAO zone. Norway is the second-largest exporter of fishery products in the world (FAO 2016), and our results suggest that it could continue to supply much of Western Europe with DHA in 2100.

Larger countries in South-East Asia along the Andaman Sea, such as Thailand and Myanmar, likewise remained above the recommended daily dose for infants. We project that DHA production in these three countries will decline by ~ 55% while their total population is projected to decline slightly (UN 2017). Conversely, the largest countries in East and South-East Asia, such as China, Japan, and Indonesia, shifted from producing a surplus of DHA in the base case to below the threshold for infants in 2100 under RCP 8.5. The populations of Japan and China are projected to decrease while the population of Indonesia is projected to increase modestly by 2100 (UN 2017), so this change is caused by the temperature-driven decline in *M*_DHA,i_ for the Northwestern Pacific and inland Asian (which includes the freshwater fisheries of Japan and Indonesia) FAO zones.

We project that all African countries except for Namibia will fall below the recommended daily dose for infants by 2100, with most of the continent producing less than 25 mg day^−1^ DHA per capita, half the lowest recommended dose for adults (Kris-Etherton et al. 2009). This was driven both by the large projected increase in population for African countries by 2100 (UN 2017) and by the temperature-driven declines in African inland and coastal fisheries DHA production. The population of Mauritania, for instance, is projected to increase ~ fourfold by 2100, while DHA production is projected to decline by ~ 55%, meaning that Mauritania will go from producing 120 to just 24 mg day^−1^ DHA per capita between the base case and 2100 under RCP 8.5.

Neither Canada nor the United States domestically produces 100 mg day^−1^ DHA per capita in either the base case or in 2100, although global imports currently help to supplement domestic DHA production, and in fact, the United States is the world’s largest importer of fish and fisheries products (FAO 2016). Russia, much of Eastern Europe, and the Indian subcontinent also do not produce more than 100 mg day^−1^ DHA per capita in either the base case or in 2100, with India and Russia both producing less than 25 mg day^−1^ DHA per capita in 2100 under RCP 8.5.

### Model uncertainty

The 90% confidence interval of the *ΣM*_DHA_ and absolute ∆*M*_DHA_ outputs varied by approximately one order of magnitude. Much of this uncertainty originated in the estimation of the current stock of DHA upon which the absolute estimates (i.e., measured in tonnes) were based, while the relative ∆*M*_DHA_ (%) varied by much less (~ 30% of the median value).

**Fig. 4:**
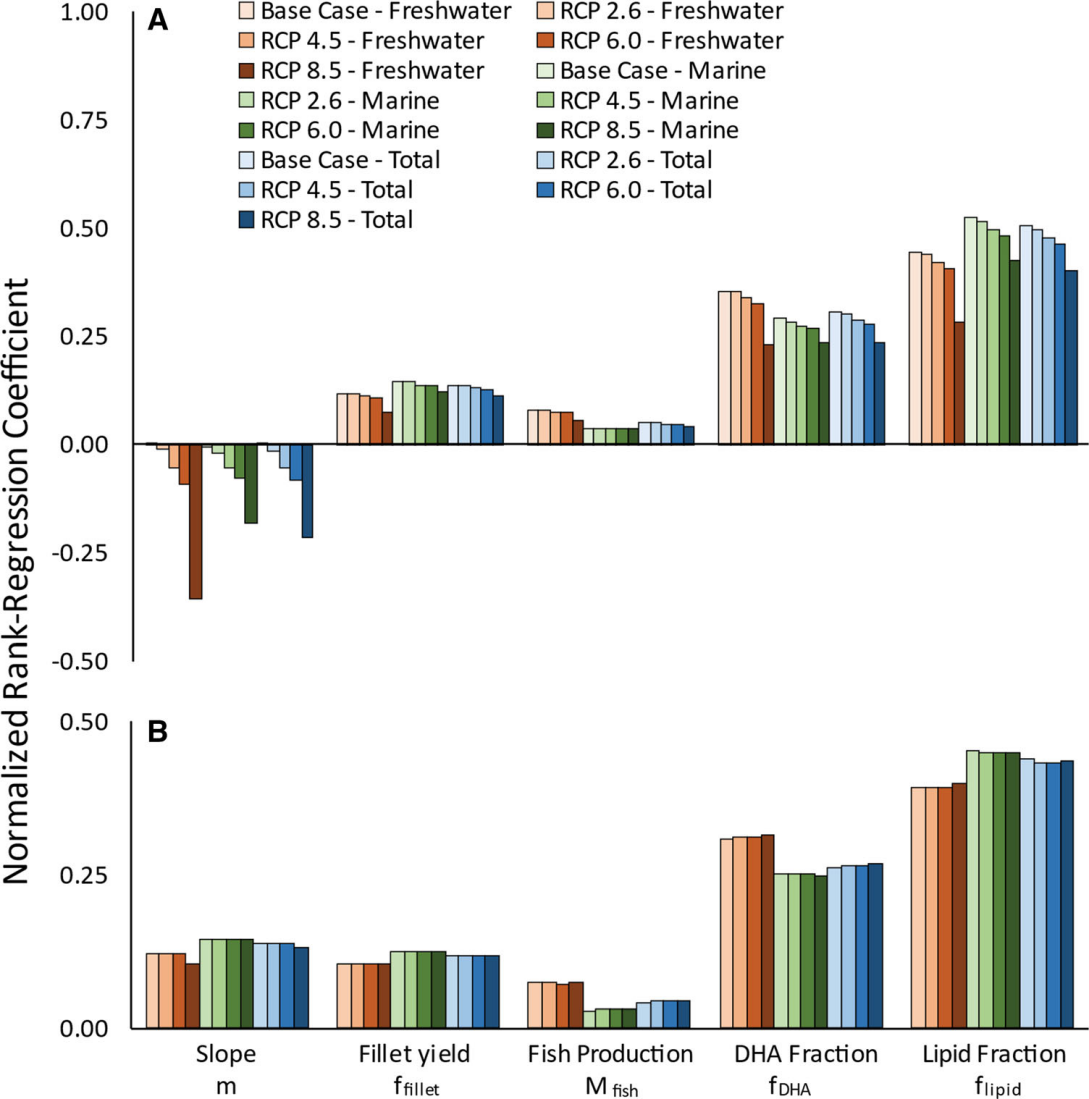
Sensitivity, as the normalized rank regression coefficient, of freshwater (red), marine (green), and total (blue) **a***M*_DHA_ and **b** ∆*M*_DHA_. The base case (shown only for *ΣM*_DHA_) is shown as the lightest shade of each color, while the projections for the year 2100 are shown in progressively darker shades of each color, with RCP 8.5 the darkest

Overall, the model was not unduly sensitive to the uncertainty of any of the individual input parameters. The largest contributions to the overall *ΣM*_DHA_ uncertainty came from *F*_lipid_, with an NRRC between 0.5 in the base case and 0.4 in RCP 8.5, followed by *F*_DHA_ with an NRRC of 0.3 in the base case and 0.2 in RCP 8.5 (Fig. 4a). These uncertainties represent the natural variability of lipid content in fish which we partially addressed by using latitudinal zones to group fish likely to be caught in the same regions. One additional way to address this uncertainty would be to use a model based on individual fish species; however, this would represent a stricter assumption that fish remained in the same FAO zones between the base case and the year 2100. Our approach indirectly accounts for the impacts of changing fish populations over time, as a major impact on DHA production from fish populations changing their geographic range in response to climate stress would be on the *F*_lipid,*i*_ and *F*_DHA,*i*_ for each FAO zone. For example, if tropical fish (with lower average *F*_lipid,*i*_ and *F*_DHA,*i*_) moved to temperate regions (with higher average *F*_lipid,*i*_ and *F*_DHA,*i*_), the average *F*_lipid,*i*_ and *F*_DHA,*i*_ for temperate FAO zones would be reduced. Our approach shows this uncertainty without needing to explicitly model the movement of fish in response to climate stress, which is not a well-understood process (Fernandes et al. 2013). The NRRC of fish production was the lowest, at around 0.05 for all scenarios.

Uncertainty in the slope of the regression describing DHA decline with temperature (*m*) provided a larger contribution to *ΣM*_DHA_ uncertainty under more severe warming scenarios, with an NRRC ranging from − 0.02 to − 0.21 under RCP 2.6 and RCP 8.5, respectively. The NRRC of m was largest for the freshwater regions in all scenarios, reflecting the larger projected temperature increases.

The contribution to the overall ∆*M*_DHA_ uncertainty followed a similar pattern (Fig. 4b), with the largest uncertainty contribution coming from *f*_lipid_ and *f*_DHA_. The contribution to uncertainty from the value of m did not depend on the climate scenario, with an NRRC of ~ 0.13 for all cases. The strong correlation between large values of m and low *ΣM*_DHA_, indicated by a high NRRC, was driven by model runs with lower *M*_DHA,*i*_ in the base case, which, when combined with high m values and large temperature changes between the base case and 2100 (*T*_2_ − *T*_1_), reached the modeled lower threshold *M*_DHA,*i*_ of zero in 2100.

## Discussion

The current amount of DHA available from fish for human consumption was estimated as ~ 370 × 10^3^ (110 × 10^3^–1500 × 10^3^ 90% confidence interval) tonnes annually. The 90% confidence interval encompasses the estimate of Tocher (2015), who used a different method to arrive at 840 × 10^3^ tonnes of EPA + DHA globally available from fish; or about twice the value of our estimate for DHA alone.

DHA production in the base case was generally highest in areas with the highest fish production, emphasizing the importance shown in other work of fisheries declines caused by climate change and overfishing (e.g., Salem and Eggersdorfer 2015; Golden et al. 2016) on the availability of DHA for human consumption. However, DHA production in equatorial regions such as Africa, South America, and the East-Central Atlantic, was notably lower than would be predicted from fish catch alone. For instance, the East-Central Atlantic had the fourth-largest fish production but was ranked eighth by DHA production. The overall fraction of DHA per tonne of fish in the base case was 0.27% in all regions, compared to an average of 0.11% in these regions (Table 3).

**Table 3:** Total fish production from aquaculture and capture fisheries (*M*_fish,*i*_, tonnes  ear^−1^), DHA production (*M*_DHA,*i*_, tonnes year^−1^), and percentage of DHA per tonne fish (*M*_DHA,*i*_/*M*_fish,*i*_, %) for each FAO zone

FAO zone (zone number)	*M*_fish,*i*_	Base case *M*_DHA,*i*_	RCP 8.5 *M*_DHA,*i*_	Base case percent DHA per tonne fish (%)	RCP 8.5 percent DHA per tonne fish (%)
Asia-Inland waters (04)	5.0E+07	8.6E+04	1.7E+04	0.17	0.03
Pacific, Northwest (61)	1.9E+07	5.6E+04	1.5E+04	0.30	0.08
Pacific, Western Central (71)	1.5E+07	2.2E+04	1.0E+04	0.15	0.07
Atlantic, Eastern Central (34)	8.6E+06	1.3E+04	5.6E+03	0.15	0.06
Atlantic, Northeast (27)	8.4E+06	4.0E+04	1.8E+04	0.47	0.21
Pacific, Southeast (87)	7.9E+06	2.2E+04	1.3E+04	0.28	0.17
Indian Ocean, Eastern (57)	7.5E+06	2.0E+04	1.1E+04	0.27	0.15
Africa-Inland waters (01)	5.9E+06	5.2E+03	1.4E+03	0.09	0.02
Indian Ocean, Western (51)	5.7E+06	1.3E+04	7.3E+03	0.24	0.13
Pacific, Eastern Central (77)	2.5E+06	5.1E+03	2.4E+03	0.21	0.10
Pacific, Northeast (67)	2.3E+06	6.6E+03	1.4E+03	0.28	0.06
Mediterranean and Black Sea (37)	2.3E+06	7.1E+03	1.9E+03	0.31	0.08
Atlantic, Southeast (47)	1.9E+06	7.3E+03	4.4E+03	0.38	0.23
Atlantic, Southwest (41)	1.6E+06	4.3E+03	2.6E+03	0.26	0.16
America, South-Inland waters (03)	1.3E+06	1.1E+03	2.9E+02	0.09	0.02
Atlantic, Western Central (31)	1.2E+06	3.0E+03	1.3E+03	0.24	0.10
Europe-Inland waters (05)	1.1E+06	4.3E+03	1.7E+02	0.40	0.02
Atlantic, Northwest (21)	9.3E+E+05	3.4E+03	1.6E+03	0.36	0.17
America, North-Inland waters (02)	6.2E+05	2.7E+03	1.4E+02	0.44	0.02
Pacific, Southwest (81)	5.3E+05	1.6E+03	1.0E+03	0.30	0.20
Oceania-Inland waters (06)	2.8E+04	4.9E+01	1.5E+01	0.17	0.05
Indian Ocean, Antarctic and Southern (58)	1.2E+04	3.9E+01	2.5E+01	0.33	0.21
Arctic sea (18)	9.9E+03	3.4E+01	7.4E+00	0.34	0.07
Pacific, Antarctic (88)	3.5E+03	1.2E+01	1.2E+01	0.33	0.33
Atlantic, Antarctic (48)	2.7E+03	1.0E+01	6.1E+00	0.37	0.22
Antarctica-Inland waters (08)	–	–	–	–	–
Total	1.4E+08	3.2E+05	1.2E+05	0.22	0.08

Each of the IPCC RCP scenarios represent different assumptions about efforts to curtail greenhouse gas emissions, and therefore limit global warming. The RCP 2.6 scenario assumes that humans will act immediately to severely curtail emissions, while RCP 8.5 represents the “business as usual” scenario where emissions continue to increase, and the others fall in-between. Our subsequent analysis will focus on the RCP 8.5 scenario, as current efforts to combat climate change are most consistent with this pathway (Friedlingstein et al. 2014) and, even with the new targets in the Paris Agreement, large “negative” greenhouse gas emissions will soon become necessary as delays in emissions reductions continue (Sanderson et al. 2016).

Under RCP 8.5 in 2100, the disparity between areas with the highest fish catch and areas with the highest DHA production became more pronounced. The Northwest Atlantic, with the 5^th^ largest fish production, went from 3rd to the 1st highest *M*_DHA,*i*_ between the 2010 base case and 2100, while the Western Central Pacific, which has the 3rd largest fish production, dropped from the 4th to the 6th highest *M*_DHA,*i*_ over this period. Overall, the fraction of DHA per tonne of fish in 2100 under RCP 8.5 dropped as low as 0.02% in the inland fisheries of Europe, North America, South America, and Africa, due to the large projected temperature increases for inland fisheries. For marine areas, DHA production in lower latitudes and the Northern Pacific greatly declined while production from zones in the Southern Ocean, the North Atlantic, and the temperate Pacific was more stable (Fig. 2).

Freshwater fish, especially in temperate climates, were predicted to have the greatest proportional decline in DHA (Fig. 2). The fraction of DHA per tonne of fish in 2100 under RCP 8.5 was under 0.05% for the inland fisheries, vs an average of 0.08% for all fisheries. This was due to the larger predicted temperature increases in freshwater than in the oceans. Asia has the greatest catch and supply of freshwater fish (FAO 2016), and also the greatest share of the world’s population (UN 2017); therefore, the consequences of the predicted severe decline in DHA production from the Asian inland and coastal FAO zones could be great.

We estimated the total global per capita production as 140 mg day^−1^ DHA today, significantly over the threshold of 100 mg day^−1^ DHA recommended by the FAO for infants. However, given the actual distribution of DHA production, we estimated that 58% of the world’s population currently live in countries where DHA availability from domestic fish production is below this threshold. Under RCP 8.5, 96% of the global population will fall below this threshold by 2100 (Fig. 3). Golden et al. (2016) estimated that 19% of the world’s population is vulnerable to nutrient deficiencies (including DHA) due to declines in fish catch caused by overfishing and climate change. Our results, although not perfectly comparable, show that the impacts of homeoviscous adaptation alone on DHA availability will put many more than the most vulnerable at risk of DHA deficiency by 2100.

Shortages of DHA are likely to have the most impact on infants, as DHA intake is critical for the developing brain. This concern is most acute in developing nations in tropical regions, where human nutrition depends most on wild fish, and where fisheries are at most risk for declines caused by illegal fishing, poor governance, population pressures, and climate change (Golden et al. 2016). Our data suggest that the estimated availability of DHA in 2100 is lowest globally in African countries, where in 2100 under RCP 8.5, only Namibia would produce more than 100 mg day^−1^ DHA per capita, while most other African nations will produce less than 25 mg day^−1^ DHA per capita. Further, we estimated that DHA production in African inland waters could decrease by 65% due to warming alone. Thus, people inhabiting this continent, and especially those relying on inland fisheries, may be particularly vulnerable to the projected decreases in DHA.

North America and most of Western Europe fall under the threshold for sufficient per capita DHA by the year 2100, but international trade may be able to compensate for this decline, assuming current trading patterns continue in the future. Today, 78% of seafood products are sold in markets influenced by international trade competition (Tveterås et al. 2012), with Latin America, Oceania, Africa, and Asia net exporters of fish and fisheries products, while Europe and North America are net importers and consume significantly more fish than they produce (FAO 2016; Guillen et al. 2019).

Many organizations worldwide recommend at least ~ 250 mg day^−1^ DHA plus EPA, or ~ 125 mg day^−1^ for DHA alone, for adults (Cunnane et al. 2000; WHO 2008; Rimm et al. 2018; Zhang et al. 2018). For adult pregnant and lactating females, the minimum intake for optimal adult health and fetal and infant development is 300 mg day^−1^ EPA + DHA, of which at least 200 mg day^−1^ should be DHA (WHO 2008). Currently, only 5% of the world’s population lives in areas that produce more than 250 mg day^−1^ DHA per capita, and this would be reduced to < 1% of the world’s population by 2100 under RCP 8.5. As discussed above, Western Europe and North America are net importers of fish products, allowing consumers in these regions to meet their DHA requirements at the expense of fish-exporting countries in Africa, Asia, and Latin America. Even if the current recommended intake level is an overestimate, the mismatch between supply and demand will be of increasing concern as DHA production falls due to marine and freshwater temperature increases and the burgeoning human population.

While most DHA for human consumption is currently supplied from fish, alternative sources of DHA may be required to bridge the future gap between supply and demand. Shellfish (i.e., bivalves) are important contributors of DHA for human consumption, albeit in low quantities (lipid level 1–2% dry weight). However, both wild and farmed shellfish rely completely on wild algae production of DHA; therefore, our projected decline in DHA would impact shellfish DHA production as well. Microalgae have become one of the most promising and innovative sources of functional food, feed, and nutraceutical products (Matos et al. 2017), with several species of microalgae being substantial producers of DHA. Microalgae production is becoming popular as a source of DHA. There are several products now on the market that are rapidly being added as nutritional supplements and aquaculture feeds (Sprague et al. 2017; Tibbetts 2018). Phototrophic production of microalgae (i.e., using light) results in slower production but yields a combination of EPA and DHA, while heterotrophic production has high yield, but only produces DHA. Commercial scale production is building; however, at the present time it is an expensive resource, and requires high-tech infrastructure to grow. The potential production of DHA by genetically engineered terrestrial oilseeds, such as canola and camelina, shows promise to meet the human demand for DHA, however, these crops have not yet reached a mature stage of commercialization (Napier et al. 2019). It should also be considered that these terrestrial oilseeds will also compete for areal land for other food production, and there are potential ecosystem effects of introducing DHA on land (Colombo et al. 2017; Sutherland et al. 2018). Another example of transgenic production of DHA is yeast, which produces up to 5.6% DHA in the total oil fraction (Damude et al. 2009). Transgenic mammals (e.g., mice; Kang et al. 2004) have also been bred to accumulate high levels of DHA in their tissues, and which, in the case of pigs (Zhou et al. 2014), could be used as traits in livestock destined for human consumption. A summary of novel transgenic organisms that produce DHA can be found in Osmond and Colombo (2019). However, there are obvious social and ethical considerations regarding these technologies that inevitably must be addressed before harvesting DHA from such novel sources.

Further, development of alternative DHA sources can further exacerbate differential accessibility with vulnerable nations most at risk of DHA deficiencies. For example, Africa as the most vulnerable continent produces significantly less farmed fish (as a supply of DHA) than other regions (FAO 2018). However, even within regions significant differences occur, e.g., Cambodia relies primarily on wild freshwater fish, whereas Vietnam has a significant aquaculture industry (FAO 2018) leaving Cambodia more vulnerable to declining DHA. Access to new transgenic sources of DHA that are predominantly produced in high-income countries may not extend to DHA-vulnerable low-income countries. Thus, higher income countries may be able to meet a reduced DHA supply by compensating with new advances in DHA production (e.g., transgenic crops, microalgae production, etc.) which will be unlikely options in lower income countries which strongly depend on wild fish catch as their main DHA resource. Therefore, variability surrounding in economic, societal, and geographic factors on DHA availability adds uncertainty and complexity to the assessment of regional DHA availability in the future.

In addition to declining fish stocks due to overfishing (Cheung et al. 2016), eutrophication poses a major threat to global DHA availability, especially in freshwater and coastal systems. Eutrophication and warming waters can reduce DHA in fish by shifting algal species from DHA-rich taxa like diatoms and cryptophytes to cyanobacteria (Paerl and Huisman 2008) and also by reducing light penetration needed for photosynthesis (Piepho et al. 2012). The significant declines we estimate for DHA production due to homeoviscous adaptation in isolation would be compounded by these additional stressors, strengthening our prediction that global warming will result in significant, spatially discrete, decreases in the production of DHA leading to shortages of this compound in marine and freshwater fish that are, to date, the main source of this essential nutrient to humans.

We note that declines in global DHA production from fish will impact non-human predators as well, as many other vertebrates require for DHA optimal functioning (Arts et al. 2009; Calder 2015). DHA is critical for the growth and survival of secondary and tertiary consumers in the wild, in aquatic animals, and terrestrial animals that have access to aquatic food sources, such as terrestrial predators (Gladyshev et al. 2009, 2013; Twining et al. 2016). As a result, animals in natural ecosystems likely will suffer from DHA limitation when dietary DHA and synthesis capacity are low (Twining et al. 2016). It is also important to consider that Arctic species may be disproportionately affected because of estimates of greater reductions in DHA production in these regions than more temperate regions. For example, many Arctic species depend on fatty-fish diets rich in DHA, such as marine mammal predators including pinnipeds (seals, walruses), cetaceans (whales, dolphins), as well as terrestrial animals that depend on marine resources, such as aquatic birds and bears. The ecological consequences of DHA limitation may include decreased growth rates, increased exposure to predation, resource competition, elevated stress response, impacts on reproduction and fecundity, etc. Overall, DHA availability can equally impact the health and importantly, the health of the offspring of mammalian predators.

## Conclusions

Ample evidence points to the health benefits accrued to humans when adequate amounts of DHA are available in the diet, especially for fetal and infant neurodevelopment. The 0.3 million tonnes of DHA currently supplied by the world’s landed wild capture and aquaculture fisheries today falls short of the recommended intake of at least 250 mg/day of EPA + DHA for optimal nutrition, assuming that roughly half of that intake would be from DHA. Even under the most optimistic climate scenario of aggressive reductions in greenhouse gas emissions, we estimated that warming waters will cause a 10% (8.2–11%) loss of globally available DHA from wild caught and farmed marine and freshwater fish, and a loss of 58% (52–68%) by 2100 under warming conditions caused by “business as usual” greenhouse emissions. The projected per capita shortages in domestically produced DHA were severe around the world but would be felt most acutely in the many low-income countries falling well below the amount of DHA recommended for fetal development and infants. This would create a global problem for vulnerable populations that depend on local fisheries as their main source of DHA (in tropical zones for example, especially around the Equator), low-income populations (particularly in developing nations), and particularly for infants that have a higher need for DHA. Predatory mammals, especially those in polar regions, are also expected to be adversely affected by reduced DHA availability. Solutions are needed to slow the rate of DHA loss in order to protect this valuable, patchily distributed, resource for future human generations and ecosystems.

We conclude that global warming, through the underlying mechanism of homeoviscous adaptation, will act to reduce the production and subsequent transfer of essential omega-3 fatty acids from algae to fish thereby significantly reducing global stocks of the essential brain-building nutrient DHA in the human food supply chain and in ecosystems.

## Electronic supplementary material

Below is the link to the electronic supplementary material.

Supplementary material 1 (XLSX 2059 kb)

Supplementary material 2 (PDF 462 kb)
