# Supplementary material for: Projected declines in global DHA availability for human consumption as a result of global warming
Source: Ambio. 2019 Sep 12;49(4):865–80. doi: 10.1007/s13280-019-01234-6 (PMC7028814; doi:10.1007/s13280-019-01234-6)
Supplement: Supplementary file 2 — Supplementary material 2 (PDF 462 kb) [file 13280_2019_1234_MOESM2_ESM.pdf]

Ambio

Electronic Supplementary Material

Title: **Projected Declines in Global Docosahexaenoic Acid Availability for Human Consumption as a Result of Global Warming**

Stefanie M. Colombo*, Timothy F.M. Rogers*, Miriam L. Diamond, Richard P. Bazinet, and Michael T. Arts

*Authors contributed equally to this project

General Information

Further details on the calculations, model parameters, and qualitative assumptions surrounding these model terms are in the Supplementary Data File "Assumptions summary" tab. All calculations were conducted according to the 27 Major Fishing Zones defined by the Food and Agriculture Organization of the United Nations (FAO 2016).

Terms in Equation 1 Calculating Global Annual Production of DHA, M_{DHA} (tonnes · year^{-1})

Global Fish Catch (M_{Fish}, tonnes year^{-1})

Total fish production in each FAO Major Fishing Zone, $M_{Fish,i}$ (tonnes year^{-1}) from aquaculture and wild caught fisheries for all diadromous, freshwater and marine fish species was separated into capture and aquaculture fisheries. For marine capture fisheries we used the Sea Around Us catch reconstruction data, which estimates unreported landings along with reported catch (Pauly and Zeller 2015). The Sea Around Us dataset does not contain estimates of inland capture fisheries or aquaculture production, and so for these we used data from the FAO (FAO 2016). Reported landings from inland fisheries are known to greatly underestimate the total catch by at least 50% (FAO 2016), and so we multiplied reported catch by a factor between 1.0 and 3.7, using a triangular distribution with the most likely value as 1.5x the reported catch to parameterize this uncertainty. The value of 3.7 is the production-weighted average factor by which reported catch was less than estimated total catch in a study by the FAO (FAO and World Fish Center 2008).

To construct the base-case scenario, we used the production from all sources in 2014, the latest year that data was available in the Sea Around Us dataset. We estimated the uncertainty of the aquaculture and marine catch data using a normal distribution with a standard deviation equal

to that of the previous five years of catch data for each FAO zone, separately for the aquaculture and the capture fisheries

Fraction of Fillet Yield from Whole Fish (F_{Fillet})

The average fillet yields, from commercially important fish species (FAO 1989) were used to estimate the total mass of the edible portion of fish (F_{Fillet}) from the production data. This value represents a global average from a total of 4,157 fish species.

Fraction of Total Lipid in Fish (F_{Lipid}) and Fraction of DHA in Total Lipid (F_{DHA})

The fraction of total lipid in fish muscle tissue, $F_{DHA,i}$ was estimated using published data for marine (n= 240; Colombo et al. 2017) and freshwater species (n= 87; Hixson et al. 2015). The average fraction of DHA, $F_{DHA,}$ in total lipid $F_{DHA,i}$ was also estimated from Colombo et al. (2017) and Hixson et al. (2015). The species within the data sets published by Colombo et al. (2017) and Hixson et al. (2015) were not exclusively commercially-relevant, nor did they include all species included in the FAO catch landing data or the FAO fillet yield data. The data used to estimate values of F_{Lipid} and F_{DHA} representative of for marine and freshwater fish for each latitudinal band (polar, tropical and temperate), e.g., providing a value for F_{Lipid} and F_{DHA} in the marine polar, marine temperate, marine tropical, freshwater polar, freshwater temperate and freshwater tropical latitudinal band. $F_{Lipid,i}$ and $F_{DHA,i}$ in each FAO zone was then calculated as the area-weighted average of the latitudinal band F_{Lipid} and F_{DHA} . It was assumed that these fractions would remain constant from T_1 current conditions to T_2 in year 2100.

Terms in Equation 2, Calculating the Change in Mass of DHA between T_1 (current) and T_2 in year 2100, ΔM_{DHA}

Slope of linear relationship between temperature and DHA content (m)

Hixson and Arts (2016) developed a linear regression model that related water temperature to DHA content in algae. Their regression model was based on 952 fatty acid profiles from 6 major algae taxa (chlorophytes, cryptophytes, cyanobacteria, diatoms, dinophytes and haptophytes), obtained from the peer-reviewed literature, covering globally-distributed marine and freshwater algae. Each fatty acid profile was associated with the temperature in which the algae sample was collected. Here we developed a new linear model based on the fatty acid profiles (n= 453) from taxa that produce the most DHA globally (cryptophytes, diatoms, and dinophytes). Cyanobacteria, for example, were removed from the dataset as they are not known to produce DHA. The term m represents the slope or rate of change of DHA as a function of temperature, which we assume to be equivalent between algae and fish.

Temperature Change (T_2-T_1)

The average marine temperature change was calculated under the four Intergovernmental Panel on Climate Change (IPCC) Representative Concentration Pathways (RCP), RCP 2.6, 4.5, 6.0 and 8.5, using data from table SM30-4 of the Fifth IPCC Annual Report (IPCC 2014; Hoegh-Guldberg et al. 2014), to obtain a value for each of the FAO Major Fishing Areas.

The freshwater temperature increase for each FAO Major Fishing Area was assumed to be equal to the "skin temperature" increase as determined from the National Centre for Atmospheric Research (NCAR) Community Climate System Model (CCSM) (NCAR, 2004). We used the NCAR GIS Program AR5 ensemble average data for the years 2100 and 2010, downloaded as monthly averages on a 1-degree grid. From the annual average in each grid square, we calculated the temperature change under each of the IPCC scenarios as the difference between the temperature under that scenario in 2100 and the average of all the scenarios in 2010. We then averaged these temperature differences in each of the FAO zones to obtain a

temperature change under each of the IPCC scenarios for each FAO zone. This calculation assumed a homogeneous temperature within each FAO zone and a yearly average temperature that neglected seasonal variability.

We also assumed that the average annual water temperature change was represented by the annual surface skin temperature change across each inland fishing zones represented, as it has been shown that surface water temperatures tend to track ground-level air temperatures (Blakey 1966; Johnson et al., 2014; Wilby et al., 2014). We tested this assumption using the NCAR Community Land Model (CLM) Lake Temperature output, which gives projected temperatures for lakes of greater than 50 m depth around the world. The freshwater temperature changes calculated from the CLM dataset for the year 2100 were within 73 – 97% of those calculated using the skin temperature dataset under RCP 8.5, with a mean of 85%. We chose to use the skin temperature dataset to represent freshwater temperature changes rather than the CLM data since the latter was only available for RCP 8.5, and this dataset did not have an even representation of lakes across all FAO zones (Oceania, for instance, only had three points).

DHA Per Capita

The estimates of DHA available per capita were obtained using data from the medium variant of the United Nations (UN) population World Population Prospects 2017 (United Nations 2017) database. We used the population of each fishing country in 2014 and in 2100 to match with the fish production data. We calculated the fraction of the total catch in each fishing zone for the year 2014 by each fishing country and used that to allocate the model results among countries (Supplementary Data “DHA Allocation” tab). A small portion (≤0.2%) of the DHA produced overall was not allocated to a country, as it either came from an unknown fishing country or from an entity without a population estimate by the United Nations.

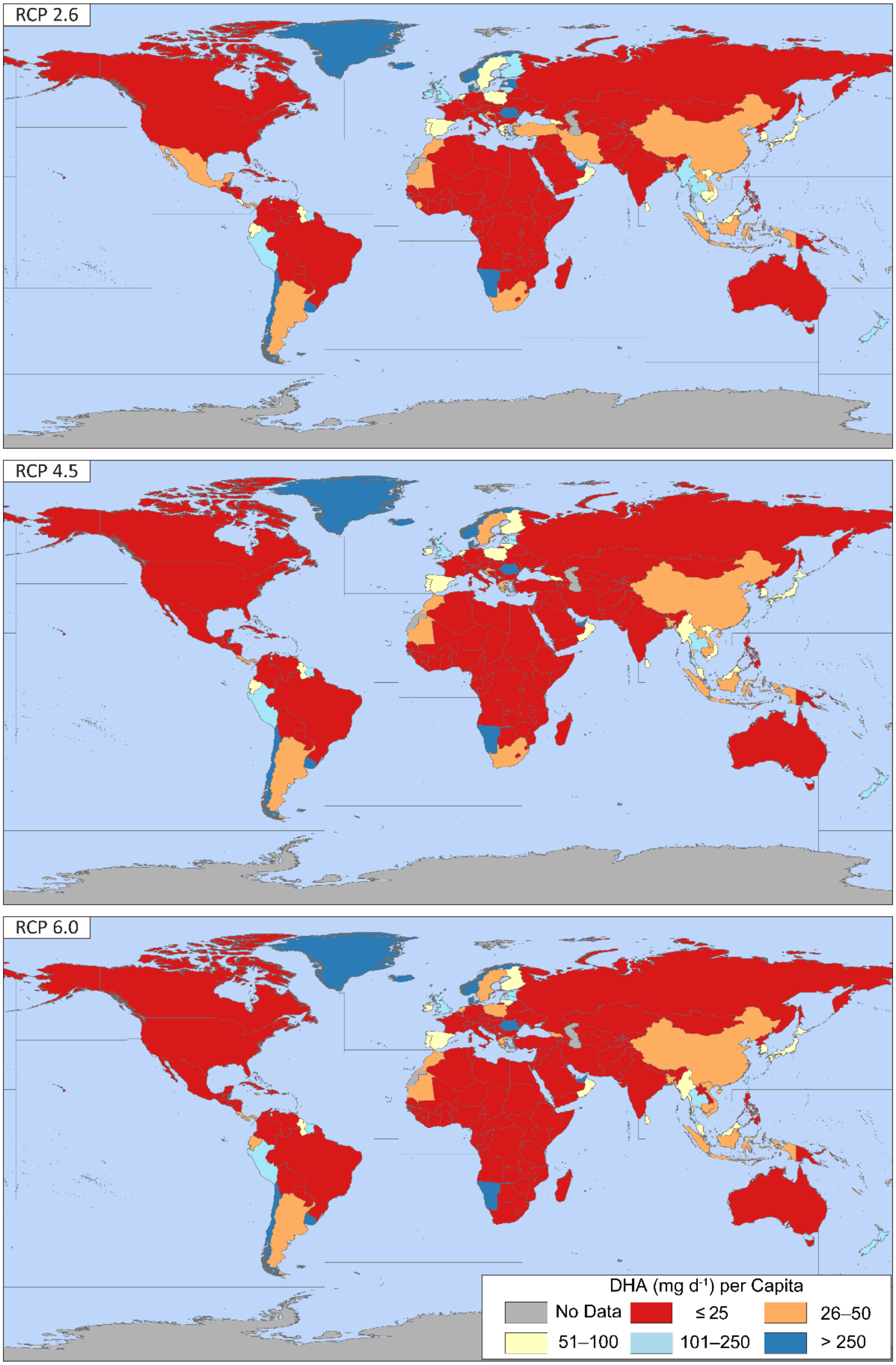

Figure S1: DHA per capita in a single fishing year by 2100, under RCP 2.6, 4.5 and 6.0. Population estimates are from the median variant of the UN World Population Prospects (2017). Political boundaries base map from https://gadm.org/.

References

Colombo SM, Parrish CC, Wacker A, Kainz MJ, Arts MT. 2017. A functional dichotomy in the production, distribution, and abundance of long-chain omega-3 polyunsaturated fatty acids between marine and terrestrial ecosystems. Environmental Reviews. 25: 163-174

FAO. 1989. Yield and nutritional value of the commercially more important fish species (Table 1). FAO Fisheries Technical Paper. Food and Agriculture Organization of the United Nations. Rome, Italy. 309: 1-187.

FAO. 2010. Fats and fatty acids in human nutrition: Report of an expert consultation. FAO Food and Nutrition. Geneva, Switzerland. http://www.fao.org/docrep/013/i1953e/i1953e00.pdf.

FAO. 2016. Part 1- World Review. In, The State of World Fisheries and Aquaculture. Food and Agriculture Organization of the United Nations. Rome Italy; 2016. pp. 2-105. URL: http://www.fao.org/fishery/statistics/global-production/query/en

FAO and World Fish Center. 2008. FAO Rome; WFC, Penang; World Bank, Washington D.C, Small-Scale Capture Fisheries: A Global Overview with Emphasis on Developing Countries.

Pauly, D., and D. Zeller. 2015. "Sea Around Us Concepts, Design and Data." seaaroundus.org.

Hixson SM, Arts MT. 2016. Climate warming is predicted to reduce omega-3 long chain polyunsaturated fatty acid production in algae. Global Change Biol. 22: 2744-2755

Hixson SM, Sharma B, Kainz MJ, Wacker A, Arts MT. 2015. Production, distribution, and abundance of long-chain omega-3 polyunsaturated fatty acids: A functional dichotomy between freshwater and terrestrial ecosystems. Environmental Reviews. 23: 414-424

Hoegh-Guldberg O, Cai R, Poloczanska ES, Brewer PG, Sundby S, Hilmi K, Fabry VJ, Jung S. 2014. The ocean - supplementary material. In: Climate Change 2014: Impacts, Adaptation, and Vulnerability. Part B: Regional Aspects. Contribution of Working Group II to the Fifth Assessment Report of the Intergovernmental Panel on Climate Change [Barros, V.R., C.B. Field, D.J. Dokken, M.D. Mastrandrea, K.J. Mach, T.E. Bilir, M. Chatterjee, K.L. Ebi, Y.O. Estrada, R.C. Genova, B. Girma, E.S. Kissel, A.N. Levy, S. MacCracken, P.R. Mastrandrea, and L.L.White (eds.)]. Available from www.ipcc-wg2.gov/AR5 and www.ipcc.ch.

IPCC. 2014. Climate Change 2014: Synthesis Report. In: Contribution of Working Groups I, II and III to the Fifth Assessment Report of the Intergovernmental Panel on Climate Change (eds Pachauri RK, Meyer LA), pp. 151, IPCC, Geneva, Switzerland.

NCAR community. June 2004. Community Climate System Model, version 3.0. http://www.cesm.ucar.edu/models/ccsm3.0/ NCAR/UCAR. GIS data services are provided by NCAR GIS Program through Climate Change Scenarios, version 2.0, 2012. URL: http://www.gisclimatechange.org. Data accessed October 2017.

UN (United Nations), Department of Economic and Social Affairs, Population Division. 2017. World Population Prospects: The 2017 Revision, Key Findings and Advance Tables. Working Paper No. ESA/P/WP. URL: https://esa.un.org/unpd/wpp/Download/Standard/Population/

Zhang, H.-M., B. Huang, J. Lawrimore, M. Menne, Thomas M. Smith, NOAA Global Surface Temperature Dataset (NOAA Global Temp), Version 4.0.0. NOAA National Centers for Environmental Information. doi:10.7289/V5FN144H. Data accessed May 2017.
